# Digital Workflow and a New Hybrid Impression Technique in Anterior Restorations Using the BOPT Approach

**DOI:** 10.3390/dj14040205

**Published:** 2026-04-02

**Authors:** Ignacio Vázquez-Natividad, Miguel R. Pecci-Lloret, Francisco Javier Rodríguez-Lozano

**Affiliations:** 1 Private Practice, 30002 Murcia, Spain; ignaciovazqueznatividad@gmail.com; 2 Gerodontology and Special Care Dentistry Unit, Morales Meseguer Hospital, Faculty of Medicine, University of Murcia, IMIB-Arrixaca, 30008 Murcia, Spain

**Keywords:** dental prosthesis design, tooth preparation, prosthodontic, dental impression technique, dental marginal adaptation, dental restoration, temporary, esthetics, dental

## Abstract

**Background/Objectives**: The biologically oriented preparation technique (BOPT) is a vertical tooth preparation approach that eliminates a conventional finish line and positions the prosthetic margin within the gingival sulcus, aiming to promote peri-restorative soft tissue adaptation through controlled gingival remodeling. This article describes a clinical case report of a hybrid impression protocol combined with a digital workflow intended to address some of the main clinical limitations of BOPT, particularly the recording of deep subgingival margins and the transfer of the emergence profile from the provisional to the definitive restoration. **Methods**: The proposed technique combined a conventional silicone impression to obtain a complete reading of the gingival sulcus with intraoral digital scanning, complemented by extraoral scanning of the provisional restoration to reproduce its subgingival morphology within the definitive prosthetic workflow. **Results**: Within the limitations of a single clinical case with short-term follow-up, this hybrid approach showed a satisfactory esthetic outcome and favorable short-term peri-coronal soft tissue behavior. **Conclusions**: This hybrid workflow may represent a feasible clinical option for transferring the cervical contour and emergence profile to the definitive prosthesis in anterior BOPT restorations.

## 1. Introduction

The aesthetic demands in the anterior region have increased notably in recent years, forcing clinicians to combine highly aesthetic outcomes with long-term stability of periodontal and peri-implant tissues. In this context, the design of the emergence profile and the integration of the restoration with the marginal mucosa have become key elements for the success of fixed prosthodontic treatments [1].

The Biologically Oriented Preparation Technique (BOPT), described by Loi in the late 2000s, proposes a restorative approach based on vertical preparations without a defined finish line and on the ability of the soft tissues to adapt to the prosthetic contour [2,3,4]. Initially applied to teeth, this concept has been shown to promote gingival thickening and marginal stability in the medium term, with low rates of biological and technical complications [5,6]. Subsequently, the concept was transferred to implant dentistry through conical, convergent abutments without a finishing line, allowing the clinician to shape the emergence profile of the crowns and to promote a stable mucosal seal around implants [2,5,7,8].

A fundamental principle of the BOPT is that gingival and peri-implant tissues can be “guided” by the cervical morphology of the crown, both in tooth- and implant-supported prostheses. For this reason, the design of the emergence profile—including controlled buccolingual overcontouring and interproximal scalloping—is crucial, especially in the anterior region, where small variations in the cervical contour can translate into noticeable changes in papillary volume, smile line, and pink–white harmony [1,2,5].

Traditionally, emergence profile conditioning in BOPT prostheses has been carried out using an analogue workflow. This approach relies on conventional impressions, stone casts, and manual modification of the gypsum model around analogues and abutments, followed by the fabrication of provisional and definitive crowns guided by that morphology. Although this protocol is well established and provides predictable results, it involves several operator-dependent steps, chairside and laboratory time consumption, and a greater difficulty in standardizing and documenting the process [1,4].

The digitalization of dentistry has introduced new possibilities at each of these stages: intraoral scanning, computer-aided design (CAD), additive or subtractive manufacturing (CAM) of models and restorations, and improved communication between clinic and laboratory. Several studies have shown that digital impressions can produce restorations with marginal fit comparable to that obtained with conventional techniques for single crowns and more extensive prostheses, remaining within the range of misfit considered clinically acceptable [9,10,11].

In the specific field of the BOPT, a fully digital workflow has been described that reproduces the classical steps carried out on the stone model. The clinician scans the intraoral situation, digitally defines the desired cervical contour, and the technician designs the “reduction” around the implant or abutment on the virtual model before printing the prototype. This resin model with removable analogues allows verification of the path of insertion and contact points of the crowns, in a similar way to the gypsum model, but with better control over the initial design, more precise communication, and a slight reduction in overall treatment time compared with the analogue workflow [1,4,9].

Despite the rapid expansion of digital workflows, doubts and resistance remain among many clinicians, particularly in highly demanding aesthetic procedures such as BOPT treatments in the anterior region. The financial investment in intraoral scanners and software, the learning curve, potential inaccuracies associated with 3D printing, and the perception that a physical model offers more “tangible” control of the case are factors that may limit full adoption of the digital workflow [1,4,9,10,11].

The literature includes isolated comparisons between analogue and digital workflows for BOPT restorations, as well as studies on the accuracy of digital versus conventional impressions. However, there is limited information specifically focused on anterior tooth-supported rehabilitations using the BOPT concept, where emergence profile management, soft tissue stability, and aesthetic predictability are particularly critical [1,4,9].

Therefore, it is clinically relevant to analyze the clinical and prosthodontic advantages of a hybrid impression protocol combined with a digital workflow for managing anterior BOPT cases. This approach integrates a conventional silicone impression to ensure a complete and accurate recording of the gingival sulcus, together with intraoral digital scanning and extraoral scanning of the provisional restoration to precisely transfer the emergence profile and subgingival morphology to the definitive prosthesis.

The aim of this study is to clinical application of a combined analogue–digital workflow for the fabrication of an anterior tooth-supported restoration using the BOPT.

## 2. Case Report

**Figure 1:**
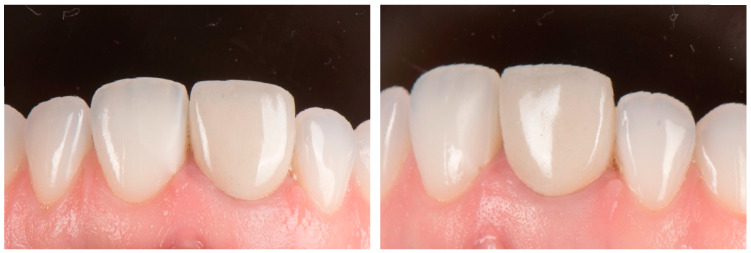
Preoperative intraoral frontal/lateral view.

A 26-year-old male patient with no relevant medical history attended a private dental clinic seeking improvement of his dental esthetics. Intraoral examination revealed a zirconia fixed dental prosthesis on the left maxillary central incisor with unsatisfactory esthetic outcomes. Clinical evaluation showed altered tooth proportions and an asymmetric gingival margin positioned more apically compared with the contralateral incisor (Figure 1). The patient presented a thick gingival biotype with inflamed peri-coronal soft tissues surrounding the affected tooth, with probing depths of 3 mm and bleeding on probing. According to the dental history, the tooth had undergone root canal treatment followed by a full-coverage restoration after trauma approximately five years earlier. The treatment plan involved the use of a vertical tooth preparation protocol based on the biologically oriented preparation technique (BOPT) to improve the gingival architecture prior to fabrication of a new zirconia crown. This case report was prepared in accordance with the CARE guidelines, and the completed checklist can be found in the Supplementary File S1.

At the initial visit, the patient was digitally scanned using an intraoral scanner (Medit i700, Medit Corp., Seoul, Republic of Korea). The digital data were used to fabricate three-dimensional printed models of the maxillary arch, which served as the basis for designing and manufacturing the provisional restoration.

**Figure 2:**
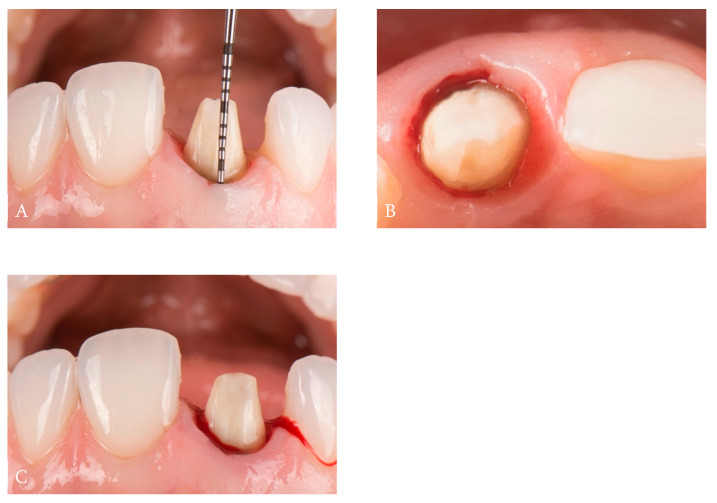
(**A**). Biologic width mapping. (**B**). Comparative detail of BOPT vertical preparation and the previous chamfer finish-line preparation. (**C**). Facial view of vertical preparation with gingival remodelation.

After removal of the existing zirconia crown, biologic width mapping was performed using a double periodontal probing technique to determine the position of the alveolar bone crest and the junctional epithelium. Vertical tooth preparation was then carried out following the protocol described by Loi, using a conical diamond rotary instrument (862.514.012 BOPT drill; Sweden & Martina, Padua, Italy) with 100/200 μm grit size and a 1.2 mm diameter, eliminating all previous coronal reference points and finish lines. Controlled bleeding was induced through sulcular preparation using the same instrumentation to initiate gingival remodeling (Figure 2).

**Figure 3:**
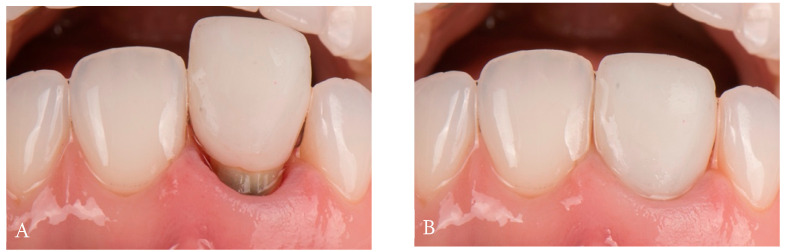
(**A**). Subcritical contour of the provisional restoration. (**B**). Provisional crown in situ showing correction and stabilization of the gingival margin.

A provisional crown was fabricated using bis-acryl resin (Protemp™ 4; 3M ESPE, St. Paul, MN, USA) and a silicone index derived from the initial model. To promote apical migration of the gingival margin, the provisional restoration was carefully contoured to generate pressure from the critical contour area, as described by González-Martín et al. [12], while positioning the crown margin within the gingival sulcus. The provisional crown was cemented using a noneugenol temporary cement (TempBond™ NE; Kerr, Orange, CA, USA) (Figure 3).

**Figure 4:**
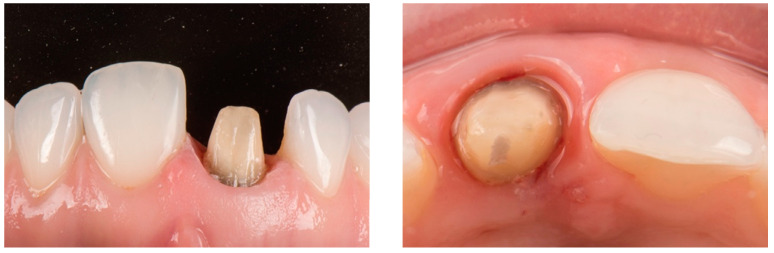
Facial and occlusal views of gingival healing, showing apical migration of the gingival margin.

After a six-week healing period, during which soft-tissue maturation was nearly complete (Figure 4), the provisional restoration was removed to allow optimal tissue stabilization prior to impression making. A double-cord retraction technique was applied using retraction cords of two diameters (Ultrapak™ #000 and #00; Ultradent Products Inc., South Jordan, UT, USA), followed by a definitive intraoral digital impression.

**Figure 5:**
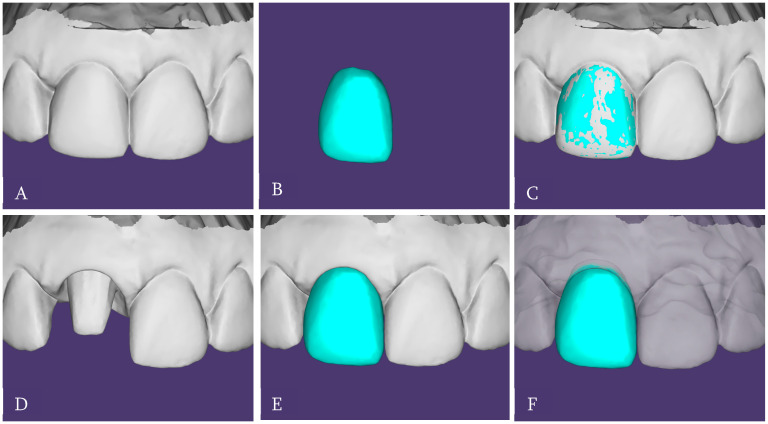
(**A**). Digital scan of interim crown in position. (**B**). Digital scan of interim crown. (**C**). Digital file of the interim crow superposed with the one in position. (**D**). Digital scan of tooth abutment resulted from hybrid impression technique. (**E**). Digital file of the interim crown in place over the tooth abutment. (**F**). Detail of the margin of the interim crown into the sulcus.

**Figure 6:**
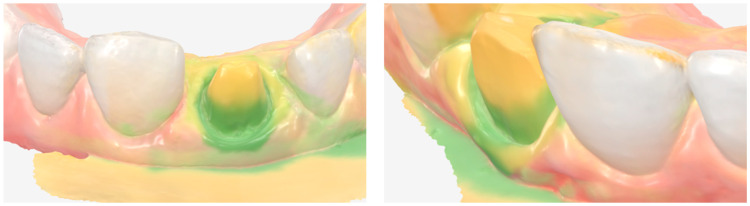
Hybrid impression technique: intraoral scan of the full arch followed by scanning of the conventional polyvinyl siloxane impression using the “scan impression” function (Medit). The impression scan was aligned with the intraoral scan through best-fit surface matching to replace the preparation area with high-resolution margin data, using the unchanged surrounding structures as reference; however, no independent quantitative validation of alignment accuracy was performed.

Additional digital scans were obtained of the provisional crown seated intraorally, in its correct maxillary position, and extraorally to capture its complete emergence profile and subgingival morphology (Figure 5). In parallel with the digital workflow, a conventional two-step polyvinyl siloxane impression of the maxillary central incisor was taken. The purpose of this analog impression was to provide a deep sulcular registration that could be integrated with the digital data to enhance margin accuracy (Figure 6). The full maxillary arch, mandibular arch, and occlusion were initially captured using an intraoral scanner (Medit i700, Medit Corp., Seoul, Republic of Korea). Subsequently, the scan data corresponding to the prepared tooth were removed from the maxillary digital model, while the remaining arch data were locked. The conventional impression was then digitized using the “scan impression” function of the Medit Link software, which converts the scanned negative impression into a positive digital model. The software automatically aligned the impression scan with the intraoral scan using a best-fit surface matching algorithm based on the unchanged surrounding structures. This process allowed the high-definition capture of the preparation margins from the impression to replace the deleted preparation area in the digital model, resulting in a hybrid dataset that combined the advantages of both digital and conventional impressions.

**Figure 7:**
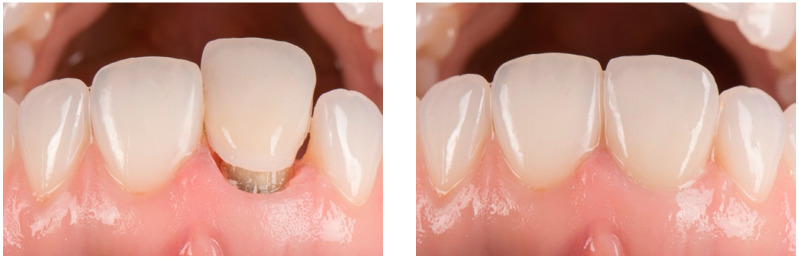
Insertion path of zirconia crown. Postoperative intraoral facial view.

Based on the combined datasets, a definitive zirconia crown with facial cutback was fabricated and layered with feldspathic ceramic. The final restoration was adhesively cemented using a self-adhesive resin cement (SpeedCEM^®^; Ivoclar Vivadent, Schaan, Liechtenstein) (Figure 7).

**Figure 8:**
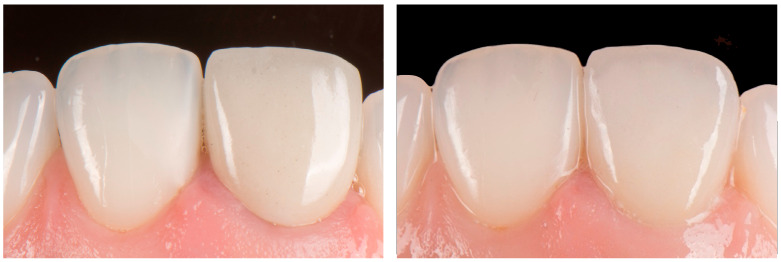
Comparison between preoperative and postoperative intraoral facial view.

During clinical evaluation at the three-month follow-up, the peri-coronal soft tissues appeared clinically healthy, with probing depths of 1 mm and absence of bleeding on probing, together with satisfactory integration of the definitive restoration (Figure 8).

## 3. Discussion

The BOPT, originally described by Loi, involves the deliberate elimination of the natural cervical contour at the cementoenamel junction (CEJ) to subsequently generate a new prosthetic emergence profile [2]. This approach seeks to achieve stable peri-restorative soft tissues over the medium and long term [13]. In BOPT, the restorative margin is intentionally placed within the sulcus, producing a controlled violation on the biologic width, as described by Loi in the original technique. Such manipulation has been associated with periodontal inflammation and potential displacement of the gingival margin [14,15]. A fundamental requirement for a favorable periodontal response is maintaining the appropriate dimensions of the biologic width and avoiding epithelial attachment invasion with the definitive prosthesis [16].

Once the CEJ or any previous finishing line has been removed, gingival conditioning is initiated. This controlled deepithelization of the sulcus stimulates fibroblast migration to the injured area [17], while the interim restoration stabilizes the formed blood clot. Because BOPT allows the gingival architecture to be shaped through the restoration’s emergence profile, the interim restoration becomes a key element in directing soft-tissue maturation [2,12]. Modifying the contour of the interim crown by adding or removing material permits fine-tuning of tissue behavior: excessive convexity tends to promote apical migration of the margin, whereas reduced contour favors coronal displacement [12].

According to Loi, the provisional margin can be positioned at different levels within the sulcus, establishing the final location of both the gingival margin and the gingival zenith [2,18]. For this reason, accurately transferring the morphology and margin depth of the provisional restoration to the laboratory is critical. Any inconsistency between provisional and definitive crowns may compromise soft-tissue stability or alter the gingival architecture obtained during provisionalization. BOPT therefore places considerable responsibility on the dental laboratory, which must reproduce margin placement and emergence profile with high precision—tasks that can be technically demanding [19].

Loi’s recommended protocol for capturing these subgingival details consists of a two-step conventional impression combined with the double-cord technique, which widens the sulcus and improves margin visibility [2]. However, intraoral scanning has become a viable alternative to traditional impressions [19,20,21]. A key limitation of digital scanning is the difficulty in accurately capturing deep subgingival margins [22]. Since complete sulcus registration is essential for correct margin placement, integrating a deep sulcular reading obtained with addition of silicones into a digital workflow helps overcome this limitation.

However, transferring the emergence profile by means of extraoral scanning of the provisional restoration alone may be insufficient if the definitive impression does not adequately capture the full depth of the gingival sulcus. In such situations, the provisional restoration may extend apically into areas of the sulcus that are not properly recorded in the final impression. This discrepancy obliges the dental technician to arbitrarily modify the cervical morphology of the definitive crown in order to adapt it to the available data, potentially altering the intended emergence profile. As a consequence, the definitive restoration may exert unintended pressure on the peri-restorative soft tissues, increasing the risk of gingival displacement and loss of the soft-tissue stability achieved during provisionalization. This limitation underscores the importance of a hybrid impression approach, in which a conventional impression ensures complete sulcular registration while digital scanning and extraoral digitization of the provisional restoration allow for an accurate transfer of the emergence profile to the definitive prosthesis.

Several authors have proposed alternative methods to facilitate the transfer of the provisional finish line and emergence profile in BOPT restorations. Bauzá et al. introduced a technique based on a custom impression coping that records both parameters [20], although the method requires prior laboratory work and is relatively time-consuming. Castelo-Baz et al. suggested placing PTFE inside the sulcus to indicate the clinician’s intended margin position [5]. While this may theoretically mark the finishing line, clinical application is hampered by difficulties in achieving stable circumferential positioning and by the material’s bulk, which alters the gingival architecture more extensively than a traditional retraction cord. Compared with these previously described approaches, the present protocol does not seek to replace them, but to provide a clinically practical alternative that combines complete sulcular registration with digital transfer of the provisional morphology within the same workflow.

Independently of the technique used, removal of the provisional restoration typically results in soft-tissue collapse, creating discrepancies between the actual gingival margin and the position recorded in the impression [19]. Various strategies have been proposed to minimize this distortion, such as custom impression copings [20] or duplicating the provisional restoration to allow the laboratory to reproduce both the margin depth and the emergence profile [3,23]. Digital workflows can streamline these procedures and enhance accuracy.

In the present case report, the gingival margin of the maxillary central incisor migrated apically during provisionalization. Rather than proposing a validated superior alternative, this report presents a clinically applicable hybrid workflow that may be useful in situations where direct intraoral scanning alone is insufficient to accurately capture subgingival morphology in anterior tooth-supported BOPT restorations. Any change in the angulation of the definitive crown’s emergence profile could relieve pressure on the gingiva and potentially induce coronaldisplacement of the margin [12]. This technical report describes a method that transfers the precise position of the provisional finish line together with the emergence profile developed during the provisional phase to the definitive cast. Rather than proposing a validated superior alternative, this report presents a clinically applicable hybrid workflow that may be useful in situations where direct intraoral scanning alone is insufficient to accurately capture subgingival morphology in anterior tooth-supported BOPT restorations. This ensures that the dental technician can accurately replicate the subgingival morphology established by the provisional restoration.

Despite the successful clinical outcome observed in this case report, several limitations merit discussion. As in any clinically applied hybrid workflow, the procedure required operator-dependent adjustments and should be interpreted as a descriptive technical demonstration rather than as an objective validation of accuracy. One of the primary technical challenges in applying a fully digital workflow to BOPT procedures is the accurate capture of deep subgingival margins. Most commercially available intraoral scanners (IOS) rely on optical technologies that require direct line of sight and adequate light reflection to generate accurate three-dimensional data. When the restorative margin is positioned within the gingival sulcus, optical access is inherently compromised by soft tissues, fluids, and limited visibility, which negatively affects scanning accuracy as margin depth increases. Previous studies have demonstrated that IOS trueness decreases significantly when the finish line is located more than approximately 0.5–1.0 mm subgingivally, potentially compromising marginal and internal adaptation unless adequate gingival retraction is performed [24,25].

Clinical studies have confirmed that the accuracy of digital impressions is negatively influenced by subgingival margin depth, and that predictable digital impressions are more difficult to achieve for deeply located margins, even under optimized clinical conditions [26,27]. Furthermore, systematic reviews have reported inconsistent accuracy of IOS for full crown preparations with subgingival margins, emphasizing the importance of effective gingival displacement and moisture control to obtain clinically acceptable results [28,29].

Although some in vitro investigations suggest that certain IOS systems may be capable of detecting vertical preparation geometries beyond the finish line at subgingival depths, these results are obtained under controlled experimental conditions and may not fully replicate the complexity of the clinical environment [30,31]. Consequently, until intraoral scanning technology achieves reliable and reproducible capture of deep subgingival anatomy, hybrid workflows combining digital and conventional impression techniques remain a valid and promising approach for ensuring accurate margin reproduction in BOPT restorations.

Future advancements in scanner hardware, image acquisition algorithms, and soft-tissue management strategies are expected to further enhance digital impression accuracy. Improvements in optical systems, alternative imaging modalities, and artificial intelligence–assisted margin detection may eventually reduce or eliminate the need for conventional impressions, enabling fully digital workflows for restorations with subgingival margins [32,33].

Another limitation of the present study is its design as a single clinical case report, which inherently restricts the generalizability of the findings. In addition, the clinical outcomes presented here should be interpreted as short-term descriptive observations rather than as standardized quantitative assessments. Therefore, the present report should be interpreted as a clinical demonstration of feasibility rather than as evidence of superiority over other impression workflows. Comparative clinical studies evaluating exclusive intraoral scanning protocols versus hybrid workflows integrating conventional and digital impressions are needed. Such investigations would allow objective assessment of the maximum sulcular depth accurately captured by each method and provide evidence-based guidelines for workflow selection in BOPT restorations.

## 4. Conclusions

The technique described above may help reduce the inaccuracy associated with arbitrarily positioning the restorative margin within the sulcus by incorporating a complete sulcular registration into the digital workflow and facilitating the transfer of the emergence profile to the dental laboratory. Within the limitations of a single clinical case with short-term follow-up, this approach may represent a feasible option for maintaining the prosthetic and biological rationale of BOPT restorations.

## 5. Diagnostic Assessment

Diagnostic assessment included clinical and radiographic evaluation. A cone-beam computed tomography (CBCT) scan was performed to rule out the presence of apical pathology and to assess the condition of the surrounding bone structures. Periodontal examination revealed a gingival probing depth of approximately 3 mm around the involved tooth, with bleeding on probing. These findings were consistent with a stable periodontal condition, allowing the planned restorative treatment using the BOPT approach.

## 6. Participant Perspective

Because the tooth involved was located in the anterior region, the patient initially expressed concern about the esthetic outcome and the clinical procedures required during treatment. However, one week after placement of the provisional restoration, the patient reported feeling more confident with the appearance of the tooth and expressed satisfaction with the improvement in esthetics.

## 7. Adverse and Unexpected Events

No biological or mechanical complications occurred during treatment. However, at the time of the definitive crown delivery, additional esthetic adjustments to the final shade were considered necessary. As a result, the placement of the definitive restoration was postponed for one week to allow refinement of the prosthesis and ensure optimal esthetic integration.

## Data Availability

The original contributions presented in this study are included in the article and Supplementary Materials. Further inquiries can be directed to the corresponding author.

## Supplementary Materials

The following supporting information can be downloaded at: https://www.mdpi.com/article/10.3390/dj14040205/s1, Supplementary File S1: CARE Checklist.
