# Supplementary material for: Digital Workflow and a New Hybrid Impression Technique in Anterior Restorations Using the BOPT Approach
Source: Dent J (Basel). 2026 Apr 2;14(4):205. doi: 10.3390/dj14040205 (PMC13115465; doi:10.3390/dj14040205)
Supplement: Supplementary file 1 [file dentistry-14-00205-s001.zip › dentistry-4146802-supplementary.pdf]

CARE
case report guidelines

CARE Checklist of information to include when writing a case report

(cc) BY-NC-ND

Topic	Item	Checklist item description	Reported on Line
Title	1	The diagnosis or intervention of primary focus followed by the words "case report"	PAGE 1 LINE 1-3
Key Words	2	2 to 5 key words that identify diagnoses or interventions in this case report, including "case report"	PAGE 1, LINES 33-37
Abstract (no references)	3a	Introduction: What is unique about this case and what does it add to the scientific literature?	PAGE 1, LINES 15-25
	3b	Main symptoms and/or important clinical findings	PAGE 1, LINES 18-28
	3c	The main diagnoses, therapeutic interventions, and outcomes	PAGE 1, LINES 18-28
	3d	Conclusion—What is the main "take-away" lesson(s) from this case?	PAGE 1, LINES 25-31
Introduction	4	One or two paragraphs summarizing why this case is unique (**may include references**)	PAGE 1 LINES 42-48 PAGE 2 LINES 4-55
Patient Information	5a	De-identified patient specific information.	PAGE 3, LINES 31-32
	5b	Primary concerns and symptoms of the patient.	PAGE 3, LINES 31-38
	5c	Medical, family, and psycho-social history including relevant genetic information	PAGE 3, LINES 31-39
	5d	Relevant past interventions with outcomes	PAGE 3, LINES 38-42
Clinical Findings	6	Describe significant physical examination (PE) and important clinical findings.	PAGE 3, LINES 32-42
Timeline	7	Historical and current information from this episode of care organized as a timeline	PAGE 12, LINES 1-17
Diagnostic Assessment	8a	Diagnostic testing (such as PE, laboratory testing, imaging, surveys).	PAGE 12, LINES 18-24
	8b	Diagnostic challenges (such as access to testing, financial, or cultural)	NOT REPORTED
	8c	Diagnosis (including other diagnoses considered)	PAGE 3, LINES 32-42
	8d	Prognosis (such as staging in oncology) where applicable	NOT REPORTED
Therapeutic Intervention	9a	Types of therapeutic intervention (such as pharmacologic, surgical, preventive, self-care)	PAGE 3, LINE 40 TO PAGE 4 LINE 13
	9b	Administration of therapeutic intervention (such as dosage, strength, duration)	PAGE 4, LINES 5-39
	9c	Changes in therapeutic intervention (with rationale)	PAGE 12, LINES 9-11
Follow-up and Outcomes	10a	Clinician and patient-assessed outcomes (if available)	PAGE 7, LINES 9-13, PAGE12, LINES 25-29
	10b	Important follow-up diagnostic and other test results	PAGE 7, LINES 9-13
	10c	Intervention adherence and tolerability (How was this assessed?)	NOT REPORTED
	10d	Adverse and unanticipated events	PAGE 12, LINES 30-35
Discussion	11a	A scientific discussion of the strengths AND limitations associated with this case report	PAGE 8, LINE 8 TO PAGE 9, LINE 53
	11b	Discussion of the relevant medical literature **with references**.	PAGE 7 LINE 21 TO PAGE 9, LINE 53
	11c	The scientific rationale for any conclusions (including assessment of possible causes)	PAGE 8, LINE 8 TO PAGE 9 , LINE 53
	11d	The primary "take-away" lessons of this case report (without references) in a one paragraph conclusion	PAGE 10, LINES 4-12
Patient Perspective	12	The patient should share their perspective in one to two paragraphs on the treatment(s) they received	PAGE 12, LINES 25-29
Informed Consent	13	Did the patient give informed consent? Please provide if requested	Yes ☒ No ☐
